# Supplementary material for: Levels of Polychlorinated Dibenzo-p-Dioxins/Furans (PCDD/Fs) and Dioxin-Like Polychlorinated Biphenyls (DL-PCBs) in Human Breast Milk in Chile: A Pilot Study
Source: Int J Environ Res Public Health. 2021 Apr 30;18(9):4825. doi: 10.3390/ijerph18094825 (PMC8125136; doi:10.3390/ijerph18094825)
Supplement: Supplementary file 1 [file ijerph-18-04825-s001.zip › ijerph-1169237-supplementary.pdf]

Supplementary Material

Table S1. Informed Detection Limit (DL) of the method and the percentage of recovery of each congener of dioxin-like polychlorinated biphenyls (DL-PCBs), polychlorinated dibenzodioxins (PCDD) and polychlorinated dibenzofurans (PCDF) analyzed (Laboratory of Veterinary Pharmacology of Universidad de Chile). The equivalent toxic concentrations (WHO-TEQ) values were calculated based on WHO Toxic Equivalency Factors (TEF) (2005) and the levels found (pg/g) in the breast milk samples.

Congener name	TEF WHO 2005	Arica				Coltauco				Molina			
		Level pg/g	% Recovery	DL pg/g	pg TEQ WHO/g	Level pg/g	% Recovery	DL pg/g	pg TEQ WHO/g	Level pg/g	% Recovery	DL pg/g	pg TEQ WHO/g
**Furans**													
2,3,7,8-TCDF	0.1	0.021	53	0.006	0.002	0.006	55	0.002	0.0006	0.007	45	0.002	0.0007
1,2,3,7,8-PeCDF	0.03	0.033	58	0.008	0.001	0.015	69	0.003	0.0005	0.016	59	0.004	0.0005
2,3,4,7,8-PeCDF	0.3	0.087	63	0.007	0.03	0.138	73	0.002	0.04	0.098	62	0.004	0.03
1,2,3,4,7,8-HxCDF	0.1	0.048	55	0.004	0.005	0.053	58	0.002	0.005	0.055	35	0.004	0.006
1,2,3,6,7,8-HxCDF	0.1	0.042	58	0.004	0.004	0.055	58	0.002	0.006	0.056	35	0.004	0.006
2,3,4,6,7,8-HxCDF	0.1	0.015	57	0.004	0.002	0.025	57	0.002	0.003	0.026	35	0.004	0.003
1,2,3,7,8,9-HxCDF	0.1	0.028	49	0.006	0.003	0.003	64	0.002	0.0003	0.005	42	0.004	0.0005
1,2,3,4,6,7,8-HpCDF	0.01	0.017	53	0.003	0.0002	0.028	60	0.001	0.0003	0.04	39	0.001	0.0004
1,2,3,4,7,8,9-HpCDF	0.01	ND	49	0.006	0.00003	0.002	62	0.002	0.00002	0.004	42	0.002	0.00004
OCDF	0.0003	ND	52	0.009	0.000001	ND	52	0.003	0.000005	ND	37	0.003	0.0000005

**Dioxins**												
2,3,7,8-TCDD	1	ND	70	0.005	0.003	0.014	72	0.002	0.01	0.005	51	0.002	0.005
1,2,3,7,8-PeCDD	1	0.038	67	0.006	0.04	0.054	72	0.003	0.05	0.045	60	0.003	0.05
1,2,3,4,7,8-HxCDD	0.1	0.026	54	0.005	0.003	0.036	53	0.002	0.004	0.03	32	0.002	0.003
1,2,3,6,7,8-HxCDD	0.1	0.095	57	0.005	0.01	0.074	57	0.002	0.007	0.107	36	0.002	0.01
1,2,3,7,8,9-HxCDD	0.1	0.016	56	0.005	0.002	0.018	55	0.002	0.002	0.033	34	0.003	0.003
1,2,3,4,6,7,8-HpCDD	0.01	0.077	56	0.004	0.0008	0.076	57	0.002	0.0008	0.123	37	0.002	0.001
OCDD	0.0003	0.281	52	0.011	0.00008	0.251	52	0.003	0.00008	0.55	37	0.003	0.0002
**PCBs**													
PCB 81	0.0003	ND	30	0.048	0.00001	ND	62	0.07	1	ND	48	0.069	0.00001
PCB 77	0.0001	ND	28	0.057	0.000003	ND	66	0.065	3	ND	52	0.067	0.000003
PCB 123	0.00003	0.265	36	0.039	0.00001	0.602	49	0.05	2	0.615	40	0.069	0.00002
PCB 118	0.00003	21.784	34	0.040	0.0007	35.142	44	0.052	1	36.936	39	0.068	0.001
PCB 114	0.00003	1.135	35	0.043	0.00004	1.685	49	0.049	5	1.904	43	0.062	0.000060
PCB 105	0.00003	5.985	36	0.032	0.0002	5.413	33	0.043	2	5.293	28	0.071	0.0002
PCB 126	0.1	0.173	29	0.04	0.02	0.168	30	0.05	0.02	0.154	25	0.085	0.02
PCB 167	0.00003	2.561	38	0.028	0.00001	3.89	50	0.043	1	3.923	41	0.067	0.001
PCB 156	0.00003	11.949	34	0.032	0.0004	15.937	47	0.05	5	17.971	39	0.071	0.0005
PCB 157	0.00003	2.022	34	0.033	0.00006	2.549	46	0.053	8	2.954	39	0.073	0.00009
PCB 169	0.03	0.062	28	0.032	0.002	0.108	26	0.036	3	ND	25	0.035	0.0005
PCB 189	0.049	0.779	34	0.028	0.00002	1.131	46	0.036	3	1.066	37	0.049	0.00003

ND = Not detectable

Table S2. Estimated daily intake (EDI) in pg TEQ/kg bw/day of dioxin-like polychlorinated biphenyls (DL-PCBs), polychlorinated dibenzodioxins (PCDD) and polychlorinated dibenzofurans (PCDF) and their sum (PCDD/F+PCB) by infants of different ages (months).

City	Age[1] / EDI[2]	PCDD/F	PCB	PCDD/F+PCB	PCDD/F+PCB (mean fat: 2.75%)
**Arica**	0 - 2 m	18.31	4.07	22.38	29.45
Fat: 2.09%	3 - 4 m	15.19	3.37	18.56	24.42
	5 - 6 m	13.76	3.06	16.81	22.12
	7 - 12 m	7.45	1.65	9.10	11.98
	13 - 24 m	5.49	1.22	6.71	8.83
**Coltauco**	0 -2 m	22.04	4.24	26.28	20.13
Fat: 3.59%	3 - 4 m	18.28	3.51	21.79	16.69
	5 - 6 m	16.56	3.18	19.74	15.12
	7 - 12 m	8.97	1.72	10.69	8.19
	13 - 24 m	6.61	1.27	7.88	6.04
**Molina**	0 - 2 m	20.18	5.02	24.07	25.66
Fat: 2.58%	3 - 4 m	16.73	4.17	20.90	22.28
	5 - 6 m	15.16	3.77	18.93	20.18
	7 - 12 m	8.21	2.04	10.25	10.92
	13 - 24 m	6.05	1.51	7.56	8.05

[1] Age in months; [2]EDI in pg TEQ/kg bw/day

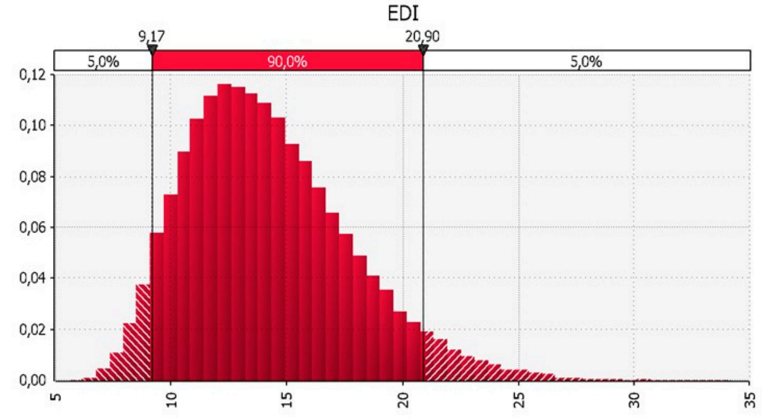

	EDI (pg TEQ/kg bw/day)
Minimum	5.57
Maximum	34.21
Mean	14.21
CI: 90%	± 0.019
Mode	13.09
Median	13.09
Std Dev	3.61
5%	9.17
25%	11.56
50%	13.75
75%	16.36
90%	19.08
95%	20.89
99%	24.53

Figure S1. Estimated daily intake (EDI) in pg TEQ/kg bw/day of the sum of dioxin-like polychlorinated biphenyls (DL-PCBs), polychlorinated dibenzodioxins (PCDD) and polychlorinated dibenzofurans (PCDF) in Chilean infants by breast milk consumption.
